# HLA Class II Genes *HLA-DRB1, HLA-DPB1*, and *HLA-DQB1* Are Associated With the Antibody Response to Inactivated Japanese Encephalitis Vaccine

**DOI:** 10.3389/fimmu.2019.00428

**Published:** 2019-03-08

**Authors:** Yufeng Yao, Huijuan Yang, Lei Shi, Shuyuan Liu, Chuanying Li, Jun Chen, Ziyun Zhou, Mingbo Sun, Li Shi

**Affiliations:** ^1^Institute of Medical Biology, Chinese Academy of Medical Sciences and Peking Union Medical College, Kunming, China; ^2^Yunnan Key Laboratory of Vaccine Research and Development on Severe Infectious Disease, Kunming, China

**Keywords:** human leukocyte antigen class II genes, HLA haplotype, inactivated Japanese encephalitis virus vaccine, antibody immune response, association

## Abstract

**Aim:** The objective of this study was to evaluate the association of the human leukocyte antigen (HLA) class II genes *HLA-DRB1, HLA-DPB1*, and *HLA-DQB1* with the humoral immune response elicited by inactivated Japanese encephalitis (JE) vaccine (IJEV).

**Methods:** A total of 373 individuals aged 3–12 years in the Inner Mongolia Autonomous Region in China, who received two doses of IJEV at 0 and 7 days, were enrolled in the current study. Based on the individuals' specific JE virus (JEV)-neutralizing antibodies (NAbs), they were divided into a seropositive and a seronegative group. *HLA-DRB1, HLA-DPB1*, and *HLA-DQB1* were genotyped using a sequencing-based typing method. Next, the association of the HLA class II genes and their haplotypes with antibody response was evaluated.

**Results:** Based on NAbs, a total of 161 individuals were classified as seropositive and 212 as seronegative. DQB1^*^02:01 was significantly associated with JEV seropositivity (*P* < 0.001, OR = 0.364, 95% CI: 0.221–0.600), while DQB1^*^02:02 was significantly associated with JEV seronegativity (*P* = 5.03 × 10^−6^, OR = 7.341, 95% CI: 2.876–18.736). The haplotypes DRB1^*^07:01-DPB1^*^04:01-DQB1^*^ 02:01, DRB1^*^15:01-DPB1^*^02:01-DQB1^*^06:02, DRB1^*^07:01-DQB1^*^02:01, and DPB1^*^02:01-DQB1^*^06:02 were very frequent in the seropositive group, while DRB1^*^07:01-DPB1^*^17:01-DQB1^*^02:02, DRB1^*^07:01-DQB1^*^02:02, and DPB1^*^17:01-DQB1^*^02:02 were very frequent in the seronegative group. The presence of DRB1^*^01:01, DRB1^*^04:05, DRB1^*^09:01, DRB1^*^12:02, DRB1^*^13:02, and DRB1^*^14:01 was associated with a higher geometric mean titer (GMT) of NAbs than that of DRB1^*^11:01 at the *DRB1* locus (*P* < 0.05). At the *DPB1* locus, the presence of DPB1^*^05:01 was associated with higher GMTs than that of DPB1^*^02:01 and DPB1^*^13:01 (*P* < 0.05), and the presence of DPB1^*^04:01 and DPB1^*^09:01 was associated with higher GMTs than that of DPB1^*^13:01 (*P* < 0.05).

**Conclusions:** The present study suggests that HLA class II genes may influence the antibody response to IJEV.

## Introduction

Japanese encephalitis (JE) is one of the most serious mosquito-borne infectious diseases, with approximately 67,900 individuals being infected by the JE virus (JEV) annually (1). Approximately 75% of these individuals are under 14 years of age, and 50% of the infections occur in China (2, 3).

Vaccination is an efficient method of controlling JEV infection. Four different types of JE vaccine are available in affected countries, namely inactivated mouse brain-derived, live attenuated cell culture-derived, inactivated cell culture-derived, and genetically engineered live attenuated chimeric vaccine. The Vero cell-derived inactivated JE vaccine (IJEV) has been widely used in China, Japan, the US, Europe, Canada, Australia, Hong Kong, Switzerland, and India (4–6). A JEV-neutralizing antibody (NAb) titer of at least 10 has been established as a correlate for protection against JEV, while positive serum conversion rate and geometric mean titer (GMT) have been used as alternative markers of efficacy of JE vaccines (7, 8). After immunization with the JE vaccine, the positive serum conversion rate ranges from 60 to 100% (1, 9). Vaccine efficacy may be influenced by factors such as the type of vaccine and the vaccinated person's age, gender, and nutritional status (6, 10). Several studies have reported that the efficacy of attenuated JE vaccine has reached 85–99.26% in Chinese, South Korean, and Nepalese children; however, it exhibited only 67.2% efficacy in Indians after primary immunization (11–14). These results indicate that different genetic backgrounds of hosts could play an important role in the efficacy of JE vaccines.

As one of the key immune gene complexes, the human leukocyte antigen (HLA) genes play an important role in the adaptive immune response to viruses and vaccines. HLA molecules are divided into three classes: class I, II, and III. Among them, HLA class II molecules (HLA-DR, -DQ, and -DP) bind to extracellular viral antigen peptides and display them on the surface of antigen-presenting cells to CD4^+^ cells to stimulate their multiplication, which, in turn, stimulate antibody-producing B cells to produce specific antibodies (15, 16). HLA genes exhibit extraordinary polymorphisms, and different alleles can affect the peptide-binding properties of the HLA molecular pocket, which subsequently influences the immune response to a vaccine. In 2005 and 2006, Ovsyannikova et al. (17, 18) observed that HLA-DPB1^*^03:01, HLA-DPB1^*^04:01, and HLA-DPB1^*^15:01 are associated with rubella vaccine-induced antibodies. On the other hand, the HLA-DRB1^*^15/16-DQB1^*^06-DPB1^*^13 haplotype has been associated with high levels of measles antibody response, but low levels of rubella antibody response.

In order to evaluate the association of HLA class II genes *HLA-DRB1, HLA-DPB1*, and *HLA-DQB1* and JEV-NAbs with the humoral immune response to IJEV, this study examined Mongolian Chinese individuals who had been administered IJEV.

## Materials and Methods

### Subjects and Vaccination

A randomized, double-blinded, positive-control, non-inferiority IJEV trial was implemented in the Inner Mongolia Autonomous Region of China from August 2012 to September 2013. The IJEV (lot: 20101201) was manufactured in a GMP-accredited facility of the Institute of Medical Biology at the Chinese Academy of Medical Sciences (IMBCAMS) and verified by the National Institute for Food and Drug Control (China, approval no. 2010L02035). Briefly, JEV P3 strains were grown on Vero cell microcarriers in a 75 L bioreactor. The virus suspension was harvested, inactivated with ultra-concentrated formalin, and purified by Sepharose 6FF and DEAE Sepharose FF. The resulting vaccine contained 0.5 mL per dose with ≥ 0.6 IU/mL JEV antigens. The clinical study procedure was approved by the Ethics Committee of the Inner Mongolia Autonomous Region Center for Disease Control and Prevention. The IJEV control (lot: 201012B02-1) was manufactured by Liaoning Chengda Biotechnology (Shenyang, China), containing the same concentration of antigens as the vaccine made by IMBCAMS. A total of 1,200 individuals aged 8 months−12 years in the Inner Mongolia Autonomous Region were enrolled to receive two doses of IJEV at 0 and 7 days. They were vaccinated with either the IJEV made by IMBCAMS or the IJEV control at a 1:1 ratio. The inclusion criteria were that the individual was in good health, was not infected by JEV, had not been inoculated with other vaccines within 7 days, and had not been inoculated with attenuated JE vaccine within 1 month. The peripheral blood samples were collected before vaccine administration and 30 days after the second dose received for the detection of neutralization antibody. Considering the limited blood sample volume and the consistency of the test, only individuals of 3–12 years of age, who were negative for NAbs before vaccination, were selected for further HLA genotyping. Finally, after vaccination, 212 individuals negative for NAbs were included in the seronegative group, and 161 individuals positive for NAbs were randomly selected and included in the seropositive group.

### Japanese Encephalitis Vaccine Neutralization Antibody Detection

JEV-specific NAbs were determined by the National Institute for Food and Drug Control using the 50% plaque-reduction neutralization test according to the requirement of the Pharmacopeia of the People's Republic of China (19). Briefly, BHK-21 cells were initially inoculated at 10^6^ cells/well in 24-well tissue culture plates and propagated for 48 h at 37°C in a CO_2_ incubator. The serum samples were inactivated for 30 min in a 56°C water bath, diluted 10-fold, and then serially diluted 2-fold from 1:10 to 1:1280 in Minimum Essential Medium (GIBCO, Grand Island, NY, USA) containing 2% fetal bovine serum and 1% penicillin/streptomycin. The diluted serum and the positive conference serum were mixed with an equal volume of diluted challenge virus (P3 strain, lot 20151102, 500 PFU/mL). The suspensions were kept in a 37°C water bath for 30 min. Afterwards, 0.1 mL aliquots of the virus-serum mixtures were dispensed separately into each well of the 24-well microplates with the BHK-21 cells. The cells were overlaid with medium containing 1% methylcellulose. The cells in the wells were stained after inoculation at 37°C for 5 days in a 5% CO_2_ incubator, and the plaques were counted. The NAb titer was defined as the reciprocal value of the last serum dilution that showed 50% or greater plaque reduction compared with the plaque counts in the virus-only control wells. NAbs at 50% plaque reduction neutralization titer (PRNT50) < 10 or PRNT_50_ increased <4-fold after vaccination were considered as negative seroconversion, while PRNT_50_ > 10, or at least a 4-fold increase after vaccination, was considered to be positive seroconversion. The antibody titers were determined by calculating the GMT as follows: GMT = Log−1 [(LogX_1_ + LogX_2_ + … LogX_n_)/n].

### *HLA-DRB1, HLA-DPB1*, and *HLA-DQB1* Genotyping

Genomic DNA was extracted from peripheral lymphocytes using the QIAamp Blood Kit (Qiagen, Hilden, Germany). *HLA-DRB1, HLA-DPB1*, and *HLA-DQB1* were genotyped using a high-resolution sequencing-based typing method (Applied Biosystems, Foster City, CA, USA). Briefly, exons 2 and 3 of *DRB1* and *DQB1* as well as all exons of *DPB1* were amplified, and the PCR products were sequenced using the BigDye Terminator v3.1 Cycle Sequencing Kit (Applied Biosystems). Finally, the sequence was analyzed with the 3730xl DNA Analyzer (Applied Biosystems), and the HLA alleles were identified using the SBTengine (Applied Biosystems).

### Statistical Analysis

The differences in age and sex between the seropositive and seronegative group were determined using Student's *t*-test or a χ^2^ test. The *HLA-DRB1, -DPB1* and *-DQB1* allele frequencies were calculated using the PyPop or PyHLA software based on the genotyping results (20–22). The Hardy-Weinberg equilibrium was assessed using the Guo and Thompson method (23). The haplotypes were constructed based on the genotyping results using the expectation-maximization algorithm (20–22). The χ^2^ test was used to determine differences in allele and haplotype frequencies between the seropositive and seronegative group. The odds ratios (ORs) and associated 95% confidence intervals (CIs) were also calculated for allele-specific risks. False discovery rate (FDR) correction was used for the multiple comparisons (20). For each gene, the amino acid sequences for all alleles were aligned together. If there was more than one amino acid at one position, a test was performed for each amino acid to examine whether it is distributed differently in the seropositive and seronegative group using PyHLA software (20). Fisher's exact test was used to analyze the association, and the odds ratio was calculated with Haldane's correction of Woolf's method (20). The association between *HLA-DRB1, -DPB1, -DQB1* alleles and antibody levels was analyzed through the analysis of variance using GraphPad Prism 7.0. *P*-values of < 0.05 were considered statistically significant.

## Results

### Characteristics of Subjects

Table 1 lists the characteristics of the enrolled subjects. They were randomly selected from the two vaccination groups, which had no difference in seroconversion or GMT (*P* > 0.05). All subjects were negative for NAbs before vaccination. After vaccination, 161 individuals with PRNT_50_ > 10 were included in the seropositive group, while 212 individuals with PRNT_50_ < 10 were included in the seronegative group. Table 2 shows that there were no age or gender differences between the seropositive and seronegative group (*P* > 0.05). In addition, there was no difference in NAbs titers according to age and gender (*P* > 0.05) in the seropositive group (data not shown). Moreover, there was no difference in HLA allele distribution according to gender (*P* > 0.05).

**Table 1:** Demographic characteristics of the IJEV NAb seropositive and seronegative group.

	**Seropositive group**	**Seronegative group**	***P*-value**
Male	75	109	0.355
Female	86	103	
Age	8.068 ± 0.201	7.611 ± 0.167	0.080

**Table 2:** Age, gender, and GMTs in the seropositive group.

	**Male**	**Female**	***P*-value**
*n*	75	86	
Anti-JEV (Log10)	1.300 ± 0.040	1.237 ± 0.035	0.237
Age	7.713 ± 0.309	8.378 ± 0.260	0.099

### Association of HLA Alleles With Neutralizing Antibody Seroconversion of Inactivated Japanese Encephalitis Vaccine

The frequencies of *HLA-DRB1, -DPB1*, and *-DQB1* were in Hardy-Weinberg equilibrium in both the seropositive and seronegative groups (*P* > 0.05). At the *HLA-DRB1* locus, the frequencies of DRB1^*^01:01 and DRB1^*^16:02 were different between the seropositive and seronegative group; however, after FDR correction, the difference was not considered significant (Table 3). At the *HLA-DPB1* locus, there was no significant difference between the seropositive and seronegative group. At the *HLA-DQB1* locus, the frequency of DQB1^*^02:01 was higher in the seropositive group (0.152) than in the seronegative group (0.061) (*P* < 0.001; OR = 0.364; 95% CI: 0.221–0.600), while the frequency of DQB1^*^02:02 was lower in the seropositive group (0.016) than in the seronegative group (0.104) (*P* = 5.03 × 10^−6^; OR = 7.341; 95% CI: 2.876–18.736) (Table 3). DQB1^*^05:01 and DQB1^*^05:02 frequencies were also different between the groups, but the difference was not significant after FDR correction (Table 3).

**Table 3:** Frequencies of HLA alleles in the IJEV NAb seropositive and seronegative group.

**Allele**	**P group**	**N group**	**P_FET**	**OR**	**95% CI**	***P***
DRB1^*^01:01	**0.047**	**0.019**	**0.034**	0.394	0.165–0.940	0.489
DRB1^*^03:01	0.065	0.061	0.880	0.936	0.517–1.696	1.000
DRB1^*^04:01	0.025	0.043	0.230	1.740	0.747–4.054	0.925
DRB1^*^04:02	0.009	0.005	0.657	0.504	0.084–3.034	1.000
DRB1^*^04:03	0.003	0.017	0.147	5.389	0.660–44.020	0.925
DRB1^*^04:04	0.019	0.009	0.342	0.502	0.140–1.793	0.925
DRB1^*^04:05	0.044	0.043	1.000	0.975	0.478–1.992	1.000
DRB1^*^04:06	0.003	0.012	0.243	3.831	0.445–32.950	0.925
DRB1^*^04:07	0.003	0.005	1.000	1.521	0.137–16.852	1.000
DRB1^*^07:01	0.124	0.125	1.000	1.007	0.649–1.562	1.000
DRB1^*^08:01	0.003	0.002	1.000	0.759	0.047–12.179	1.000
DRB1^*^08:02	0.006	0.002	0.581	0.378	0.034–4.190	1.000
DRB1^*^08:03	0.031	0.050	0.267	1.626	0.755–3.502	0.925
DRB1^*^09:01	0.130	0.123	0.824	0.932	0.603–1.440	1.000
DRB1^*^10:01	0.025	0.019	0.617	0.755	0.280–2.033	1.000
DRB1^*^11:01	0.056	0.057	1.000	1.013	0.540–1.901	1.000
DRB1^*^11:04	0.016	0.007	0.301	0.452	0.107–1.905	0.925
DRB1^*^12:01	0.075	0.057	0.367	0.745	0.415–1.338	0.925
DRB1^*^12:02	0.056	0.054	1.000	0.969	0.514–1.827	1.000
DRB1^*^13:01	0.009	0.019	0.366	2.045	0.538–7.770	0.925
DRB1^*^13:02	0.034	0.028	0.674	0.824	0.359–1.891	1.000
DRB1^*^13:03	0.003	0.012	0.243	3.831	0.445–32.950	0.925
DRB1^*^14:01	0.022	0.035	0.383	1.650	0.665–4.096	0.925
DRB1^*^14:03	0.012	0.009	0.732	0.757	0.188–3.051	1.000
DRB1^*^14:05	0.009	0.014	0.739	1.526	0.379–6.150	1.000
DRB1^*^15:01	0.099	0.101	1.000	1.023	0.631–1.657	1.000
DRB1^*^15:02	0.034	0.024	0.504	0.683	0.286–1.628	1.000
DRB1^*^15:04	0.003	0.002	1.000	0.759	0.047–12.179	1.000
DRB1^*^16:02	**0.003**	**0.026**	**0.016**	8.550	1.098–66.568	0.469
DPB1^*^02:01	0.227	0.205	0.529	0.881	0.620–1.252	0.992
DPB1^*^02:02	0.044	0.071	0.157	1.675	0.873–3.214	0.681
DPB1^*^03:01	0.065	0.045	0.252	0.672	0.355–1.273	0.818
DPB1^*^04:01	0.152	0.160	0.839	1.064	0.714–1.587	0.992
DPB1^*^04:02	0.109	0.101	0.809	0.926	0.577–1.483	0.992
DPB1^*^05:01	0.267	0.276	0.804	1.046	0.755–1.449	0.992
DPB1^*^09:01	0.037	0.014	0.053	0.371	0.138–0.999	0.681
DPB1^*^13:01	0.028	0.033	0.832	1.188	0.508–2.779	0.992
DPB1^*^14:01	0.025	0.014	0.415	0.563	0.194–1.640	0.992
DPB1^*^17:01	0.031	0.054	0.151	1.790	0.839–3.815	0.681
DPB1^*^19:01	0.006	0.007	1.000	1.140	0.189–6.864	1.000
DPB1^*^21:01	0.003	0.007	0.638	2.287	0.237–22.094	0.992
DPB1^*^41:01	0.003	0.002	1.000	0.759	0.047–12.179	1.000
DQB1^*^02:01	**0.152**	**0.061**	**6.67E-05**	**0.364**	**0.221–0.600**	**<0.001**
DQB1^*^02:02	**0.016**	**0.104**	**3.36E-07**	**7.341**	**2.876–18.736**	**5.03E-06**
DQB1^*^03:01	0.245	0.248	1.000	1.013	0.723–1.417	1.000
DQB1^*^03:02	0.044	0.057	0.502	1.320	0.672–2.594	0.845
DQB1^*^03:03	0.149	0.139	0.752	0.923	0.611–1.393	0.901
DQB1^*^04:01	0.047	0.040	0.717	0.855	0.420–1.739	0.901
DQB1^*^04:02	0.012	0.005	0.411	0.377	0.069–2.070	0.845
DQB1^*^05:01	**0.081**	**0.040**	**0.025**	0.476	0.253–0.892	0.095
DQB1^*^05:02	**0.022**	**0.057**	**0.025**	2.700	1.149–6.347	0.095
DQB1^*^05:03	0.022	0.040	0.209	1.880	0.770–4.588	0.627
DQB1^*^06:01	0.071	0.078	0.781	1.097	0.631–1.908	0.901
DQB1^*^06:02	0.093	0.078	0.507	0.822	0.490–1.378	0.845
DQB1^*^06:03	0.009	0.019	0.366	2.045	0.538–7.770	0.845
DQB1^*^06:04	0.016	0.014	1.000	0.910	0.275–3.009	1.000
DQB1^*^06:09	0.019	0.014	0.771	0.756	0.242–2.366	0.901

*P group, seropositive group; N group, seronegative group. Bold value indicated the alleles showed difference between P and N group before and after FDR correction*.

Further analysis of HLA residue levels showed that some HLA residues were associated with JEV antibody seroconversion (Supplementary Table 1). At the *HLA-DPB1* locus, residues A56, R96, T170, and V265 were associated with seronegative JEV-NAbs. At the *HLA-DQB1* locus, the residues S57, V116, A125, G135, and P146 were associated with seronegative NAbs, while V89 was associated with seropositive NAbs. At the *HLA-DRB1* locus, D28 and Y30 were associated with seronegative, while L11, K12, F13, L26, C30, I31, and Y32 were associated with seropositive NAbs. Residues containing HLA-DQB1^*^02:02 were associated with seropositive JEV-NAbs.

### Association of HLA-DRB1, -DPB1, and -DQB1 Haplotypes With Neutralizing Antibody Seroconversion of Inactivated Japanese Encephalitis Vaccine

The *HLA-DRB1, -DPB1*, and *-DQB1* alleles with frequencies higher than 0.020 in either the seropositive or seronegative group are listed in Table 4. At the level of the three loci, the frequency of the haplotypes DRB1^*^07:01-DPB1^*^04:01-DQB1^*^02:01 and DRB1^*^15:01-DPB1^*^02:01-DQB1^*^06:02 was higher in the seropositive group than in the seronegative group (*P* < 0.05), while the frequency of the haplotype DRB1^*^07:01-DPB1^*^17:01-DQB1^*^02:02 was higher in the seronegative group than in the seropositive group (*P* < 0.05). At the level of the two loci, the frequency of the haplotypes DRB1^*^07:01-DQB1^*^02:01 and DPB1^*^02:01-DQB1^*^06:02 was higher in the seropositive group (*P* < 0.05), while that of the haplotypes DRB1^*^07:01-DQB1^*^02:02 and DPB1^*^17:01-DQB1^*^02:02 was higher in the seronegative group (*P* < 0.05).

**Table 4:** Frequencies of HLA haplotypes in the IJEV NAb seropositive and seronegative group.

**Haplotype**	**P group**	**N group**	***P***	**OR**	**95% CI**	***P***
**HLA DRB1-DPB1-DQB1**						
DRB1*03:01-DPB1*02:01-DQB1*02:01	0.030	0.010	0.053	0.341	0.109–1.065	>0.05
DRB1*03:01-DPB1*04:01-DQB1*02:01	0.013	0.032	0.098	2.448	0.820–7.313	>0.05
DRB1*04:05-DPB1*05:01-DQB1*04:01	0.030	0.024	0.610	0.794	0.326–1.935	>0.05
DRB1*07:01-DPB1*04:01-DQB1*02:01	**0.046**	**0.000**	**< 0.001**	**2.380**	**2.186–2.292**	**<0.05**
DRB1*07:01-DPB1*04:01-DQB1*02:02	0.000	0.025	0.004	1.779	1.669–1.897	0.056
DRB1*07:01-DPB1*17:01-DQB1*02:02	**0.000**	**0.034**	**0.001**	**1.786**	**1.675–1.905**	**0.015**
DRB1*09:01-DPB1*02:01-DQB1*03:03	0.031	0.031	0.956	1.024	0.442–2.370	>0.05
DRB1*09:01-DPB1*04:02-DQB1*03:03	0.019	0.020	0.921	1.055	0.372–2.992	>0.05
DRB1*09:01-DPB1*05:01-DQB1*03:03	0.062	0.034	0.075	0.538	0.270–1.075	>0.05
DRB1*11:01-DPB1*02:01-DQB1*03:01	0.028	0.009	0.059	0.335	0.102–1.100	>0.05
DRB1*11:01-DPB1*04:02-DQB1*03:01	0.013	0.024	0.313	1.785	0.571–5.578	>0.05
DRB1*12:01-DPB1*05:01-DQB1*03:01	0.036	0.029	0.619	0.813	0.360–1.839	>0.05
DRB1*12:02-DPB1*05:01-DQB1*03:01	0.026	0.032	0.625	1.241	0.521–2.953	>0.05
DRB1*15:01-DPB1*02:01-DQB1*06:02	**0.029**	**0.000**	**< 0.001**	**2.355**	**2.166–2.562**	**<0.05**
DRB1*15:01-DPB1*04:01-DQB1*06:02	0.015	0.028	0.222	1.925	0.661–1.405	>0.05
DRB1*15:01-DPB1*05:01-DQB1*06:02	0.036	0.032	0.723	0.866	0.390–1.922	>0.05
DRB1*15:02-DPB1*04:01-DQB1*06:01	0.025	0.011	0.142	0.430	0.135–1.368	>0.05
**HLA DRB1-DPB1**						
DRB1*03:01-DPB1*02:01	0.029	0.017	0.252	0.566	0.211–1.519	>0.05
DRB1*03:01-DPB1*04:01	0.017	0.031	0.207	1.901	0.689–5.241	>0.05
DRB1*04:05-DPB1*05:01	0.027	0.028	0.888	1.066	0.439–2.587	>0.05
DRB1*07:01-DPB1*04:01	0.049	0.029	0.168	0.588	0.275–1.260	>0.05
DRB1*07:01-DPB1*17:01	0.012	0.036	0.037	3.109	1.012–9.549	0.629
DRB1*09:01-DPB1*02:01	0.034	0.028	0.694	0.845	0.365–1.955	>0.05
DRB1*09:01-DPB1*04:02	0.016	0.020	0.670	1.268	0.425–3.779	>0.05
DRB1*09:01-DPB1*05:01	0.064	0.040	0.172	0.634	0.328–1.225	>0.05
DRB1*11:01-DPB1*02:01	0.028	0.011	0.091	0.387	0.123–1.213	>0.05
DRB1*11:01-DPB1*04:02	0.013	0.024	0.266	1.885	0.606–5.864	>0.05
DRB1*12:01-DPB1*02:01	0.024	0.022	0.936	0.961	0.365–2.529	>0.05
DRB1*12:01-DPB1*05:01	0.034	0.023	0.407	0.692	0.288–1.661	>0.05
DRB1*12:02-DPB1*05:01	0.026	0.036	0.407	1.433	0.610–3.367	>0.05
DRB1*15:01-DPB1*02:01	0.028	0.022	0.651	0.808	0.320–2.039	>0.05
DRB1*15:01-DPB1*04:01	0.019	0.023	0.662	1.256	0.452–3.487	>0.05
DRB1*15:01-DPB1*05:01	0.040	0.041	0.873	1.062	0.507–2.225	>0.05
DRB1*15:02-DPB1*04:01	0.024	0.016	0.405	0.644	0.227–1.830	>0.05
**HLA DRB1-DQB1**						
DRB1*01:01-DQB1*05:01	0.037	0.019	0.137	0.509	0.206–1.260	>0.05
DRB1*03:01-DQB1*02:01	0.062	0.057	0.814	0.929	0.504–1.455	>0.05
DRB1*04:01-DQB1*03:01	0.022	0.035	0.253	1.692	0.681–4.200	>0.05
DRB1*04:05-DQB1*04:01	0.040	0.035	0.771	0.894	0.419–1.906	>0.05
DRB1*07:01-DQB1*02:01	**0.087**	**0.000**	**< 0.001**	**2.408**	**2.207–2.628**	**<0.05**
DRB1*07:01-DQB1*02:02	**0.016**	**0.099**	**< 0.001**	**7.158**	**2.798–18.311**	**<0.05**
DRB1*07:01-DQB1*03:03	0.016	0.021	0.541	1.409	0.468–4.245	>0.05
DRB1*08:03-DQB1*06:01	0.028	0.045	0.207	1.673	0.747–3.749	>0.05
DRB1*09:01-DQB1*03:03	0.127	0.118	0.789	0.941	0.606–1.464	>0.05
DRB1*10:01-DQB1*05:01	0.025	0.019	0.610	0.773	0.287–2.083	>0.05
DRB1*11:01-DQB1*03:01	0.053	0.052	0.983	1.007	0.525–1.929	>0.05
DRB1*12:01-DQB1*03:01	0.068	0.054	0.473	0.802	0.439–1.467	>0.05
DRB1*12:02-DQB1*03:01	0.050	0.054	0.725	1.125	0.584–2.167	>0.05
DRB1*14:01-DQB1*05:02	0.009	0.026	0.089	2.902	0.803–10.491	>0.05
DRB1*15:01-DQB1*06:02	0.090	0.073	0.455	0.818	0.482–1.388	>0.05
DRB1*15:02-DQB1*06:01	0.031	0.021	0.429	0.693	0.278–1.727	>0.05
DRB1*16:02-DQB1*05:02	0.003	0.023	0.022	7.701	0.978–60.651	>0.05
**HLA DPB1-DQB1**						
DPB1*02:01-DQB1*02:01	0.039	0.013	0.024	0.327	0.118–0.908	>0.05
DPB1*02:01-DQB1*03:01	0.063	0.046	0.342	0.735	0.388–1.390	>0.05
DPB1*02:01-DQB1*03:02	0.023	0.036	0.277	1.631	0.670–3.973	>0.05
DPB1*02:01-DQB1*03:03	0.027	0.042	0.229	1.657	0.722–3.802	>0.05
DPB1*02:01-DQB1*05:01	0.027	0.018	0.444	0.681	0.254–1.828	>0.05
DPB1*02:01-DQB1*06:02	**0.032**	**0.000**	**< 0.001**	**2.329**	**2.142–2.533**	**<0.05**
DPB1*02:02-DQB1*03:01	0.020	0.016	0.685	0.798	0.269–2.372	>0.05
DPB1*04:01-DQB1*02:01	0.058	0.032	0.097	0.551	0.270–1.125	>0.05
DPB1*04:01-DQB1*02:02	0.000	0.026	0.003	1.799	1.686–1.920	0.060
DPB1*04:01-DQB1*03:01	0.022	0.032	0.394	1.485	0.595–3.709	>0.05
DPB1*04:01-DQB1*06:01	0.027	0.018	0.399	0.657	0.245–1.759	>0.05
DPB1*04:01-DQB1*06:02	0.015	0.025	0.330	1.700	0.577–5.011	>0.05
DPB1*04:02-DQB1*03:01	0.038	0.041	0.785	1.109	0.528–2.327	>0.05
DPB1*04:02-DQB1*03:03	0.032	0.022	0.453	0.710	0.289–1.744	>0.05
DPB1*05:01-DQB1*03:01	0.077	0.089	0.488	1.206	0.710–2.048	>0.05
DPB1*05:01-DQB1*03:03	0.072	0.032	0.016	0.441	0.222–0.874	0.288
DPB1*05:01-DQB1*04:01	0.038	0.020	0.139	0.515	0.211–1.258	>0.05
DPB1*05:01-DQB1*05:02	0.000	0.026	0.003	1.799	1.686–1.920	0.057
DPB1*05:01-DQB1*05:03	0.010	0.026	0.120	2.558	0.751–8.707	>0.05
DPB1*05:01-DQB1*06:02	0.035	0.037	0.822	1.094	0.501–2.388	>0.05
DPB1*13:01-DQB1*03:03	0.012	0.020	0.411	1.646	0.496–5.465	>0.05
DPB1*17:01-DQB1*02:02	**0.000**	**0.035**	**0.001**	**1.807**	**1.692**–**1.929**	**0.021**

*P group, seropositive group; N group, seronegative group. Bold value indicated the haplotypes showed difference between P and N group before and after FDR correction*.

### Association of HLA Alleles With Neutralizing Antibody GMTs of Inactivated Japanese Encephalitis Vaccine in Seropositive Group

**Figure 1:**
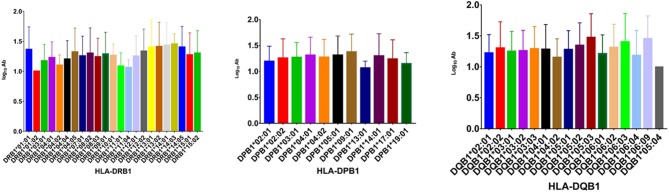
GMTs associated with different *HLA-DRB1, -DPB1*, and *-DQB1* alleles in the IJEV NAb-positive group.

To analyze the association of HLA alleles with JEV-specific NAb GMTs, the 161 individuals in the seropositive group were examined. At the *DRB1* locus, the highest GMTs were in subjects with the DRB1^*^14:03 (1.452 ± 0.174), DRB1^*^14:01 (1.430 ± 0.383), and DRB1^*^13:02 (1.410 ± 0.410) alleles, while the lowest were in those with DRB1^*^01:01 (1.000 ± 0), DRB1^*^11:04 (1.060 ± 0.135), and DRB1^*^11:01 (1.084 ± 0.226) alleles. At the *DPB1* locus, subjects with DPB1^*^09:01 (1.376 ± 0.343), DPB1^*^05:01 (1.315 ± 0.369), and DPB1^*^04:01 (1.313 ± 0.347) alleles had higher GMTs than those with DPB1^*^13:01 (1.067 ± 0.133), DPB1^*^19:01 (1.151 ± 0.213), and DPB1^*^02:01 (1.198 ± 0.289) alleles *(P* < 0.05). At the *DQB1* locus, the highest GMTs were in subjects with the DQB1^*^05:03 (1.473 ± 0.383), DQB1^*^06:09 (1.452 ± 0.369), and DQB1^*^06:03 (1.401 ± 0.460) alleles, while the lowest were in those with DQB1^*^05:04 (1.000 ± 0), DQB1^*^04:02 (1.151 ± 0.301), and DQB1^*^06:04 (1.181 ± 0.404) alleles (Figure 1).

When the alleles were compared one by one, some were associated with higher GMTs (Table 5). The presence of DRB1^*^01:01, DRB1^*^04:05, DRB1^*^09:01, DRB1^*^12:02, DRB1^*^13:02, and DRB1^*^14:01 was associated with higher NAb GMTs than the presence of DRB1^*^11:01 (*P* < 0.05) at the *DRB1* locus. At the *DPB1* locus, the presence of DPB1^*^05:01 was associated with higher GMTs than the presence of DPB1^*^02:01 and DPB1^*^13:01 (*P* < 0.05), and the presence of DPB1^*^04:01 and DPB1^*^09:01 was associated with higher GMTs than the presence of DPB1^*^13:01 (*P* < 0.05). There was no significant difference in the GMT between the different *DQB1* alleles (*P* > 0.05).

**Table 5:** GMTs of JEV NAbs associated with HLA alleles.

**Alleles**		**Allele No**.	**GMTs**	***P*-value**
DRB1*01:01 vs. DRB1*11:01	DRB1*01:01	15	1.361 ± 0.098	0.020
	DRB1*11:01	18	1.084 ± 0.053	
DRB1*04:05 vs. DRB1*11:01	DRB1*04:05	14	1.323 ± 0.107	0.049
	DRB1*11:01	18	1.084 ± 0.053	
DRB1*09:01 vs. DRB1*11:01	DRB1*09:01	12	1.287 ± 0.056	0.034
	DRB1*11:01	18	1.084 ± 0.053	
DRB1*11:01 vs. DRB1*12:02	DRB1*11:01	18	1.084 ± 0.053	0.027
	DRB1*12:02	18	1.334 ± 0.087	
DRB1*11:01 vs. DRB1*13:02	DRB1*11:01	18	1.084 ± 0.053	0.012
	DRB1*13:02	11	1.410 ± 0.124	
DRB1*11:01 vs. DRB1*14:01	DRB1*11:01	18	1.084 ± 0.053	0.022
	DRB1*14:01	7	1.430 ± 0.145	
DPB1*02:01 vs. DPB1*05:01	DPB1*02:01	73	1.198 ± 0.034	0.029
	DPB1*05:01	86	1.315 ± 0.040	
DPB1*04:01 vs. DPB1*13:01	DPB1*04:01	49	1.313 ± 0.050	0.044
	DPB1*13:01	9	1.067 ± 0.044	
DPB1*05:01 vs. DPB1*13:01	DPB1*05:01	86	1.315 ± 0.040	0.036
	DPB1*13:01	9	1.067 ± 0.044	
DPB1*09:01 vs. DPB1*13:01	DPB1*09:01	12	1.376 ± 0.099	0.038
	DPB1*13:01	9	1.067 ± 0.044	

## Discussion

Vaccines are one of the greatest advances in controlling infectious diseases in the past 300 years. The humoral immune response induced by a vaccine produces NAbs, so the seroconversion rate and GMT are widely used to evaluate vaccine efficacy. In the present study, we examined the association of HLA class II genes with the IJEV antibody response to reveal the role of the genetic variation in the HLA class II genes in the IJEV immune response.

HLA class II molecules present viral antigens in the form of peptides derived from the extracellular processing of vaccine peptides, which plays an important role in the humoral immune response to vaccines (24, 25). In an inactivated vaccine, the extracellular vaccine antigens are degraded into smaller peptides and integrated with the HLA class II molecule to constitute the HLA class II peptide complex, which plays a major role in stimulating the differentiation of CD4^+^ T cells into Th1 and Th2 cells; in turn, the Th2 cells can interact with B cells to promote differentiation into antibody-secreting plasma cells, thus secreting a specific antibody against the vaccine antigen (18).

To date, many studies have reported that HLA class II genes are associated with the vaccine antibody response (17, 26–30). In 1999, McDermott et al. reported that DQB1^*^02:02 was associated with a negative antibody response to hepatitis B virus (HBV) vaccination in a population in England (31). In 2005, Ovsyannikova et al. performed a study of the association between HLA and the humoral immune response to measles-mumps-rubella vaccination, finding that DQB1^*^02:02 was negatively associated with rubella-specific lymphoproliferation (17). In the present study, DQB1^*^02:02 was significantly negatively associated with the IJEV response (*P* = 5.03 × 10^−6^; OR = 7.341; 95% CI: 2.876–18.736). However, contrary to previous studies on the HBV, measles, rubella, influenza, and serogroup C meningococcus vaccines, which showed that DQB1^*^02:01 was negatively associated with vaccine-induced antibody response (32–34), in the present study, DQB1^*^02:01 had a significantly positive association with IJEV seropositivity (*P* < 0.05; OR = 0.364; 95% CI: 0.221–0.600). Interestingly, DQB1^*^02:01 is reportedly associated with high Th1 IFN-γ secretion, while DQB1^*^02:02 is associated with a low measles-specific Th2 cytokine response (35). There is only one amino-acid difference between DQB1^*^02:01 and DQB1^*^02:02, namely at position 135 in the peptide binding groove, where DQB1^*^02:01 contains aspartic acid and DQB1^*^02:02 contains glycine. In 2018, Yang et al. predicted the 3D ribbon models of the HLA proteins and indicated that the amino acid position 135 of HLA-DQB1 was located on the junction point of two β-sheet structures and lies on the β2 domain of protein belonging to Ig protein superfamily (36). The domain is expressed on the extracellular part of the antigen presenting cells and could integrate with CD4+ T cells during the antigen presenting process (36). Thus, we deduced that the amino acid change from a negatively charged Asp in DQB1^*^02:01 to an uncharged polar Gly in DQB1^*^02:02 could influence the JEV antigen presentation process, in consequence, the inducing of JEV-specific NAb. The further studies on the exact role of how HLA-DQB1^*^02:01 and HLA-DQB1^*^02:02 in the progression of JEV-antibody needs to be elucidated in the future.

We compared the previously reported *HLA-DQA1* and *-DQB1* haplotypes to assess whether there is any preference for DQB1^*^02:01 or DQB1^*^02:02 over *DQA1* and found that DQB1^*^02:01 and DQB1^*^02:02 are either in linkage disequilibrium with the same *DQA1* alleles, namely DQA1^*^02:01, DQA1^*^05:01, and DQA1^*^03:01 (http://www.allelefrequencies.net/). We then predicted DQB1^*^02:01 and DQB1^*^02:02 heterodimers with *DQA1* using the E protein sequence of JEV by NetMHCIIpan (http://www.cbs.dtu.dk/services/NetMHCIIpan/logos.php). The predicted peptides of DQA1^*^03:01-DQB1^*^02:01 and DQA1^*^03:01-DQB1^*^02:02 showed no difference. The identification of the actual *DQA1-DQB1* haplotypes existing in the Mongolian population would help for validation of the *DQA1-DQB1* molecular binding with specific JEV epitopes in the future.

In 2012, Schillie et al. found that DRB1^*^13:01 and DRB1^*^13:02, with an allele difference at position 86, showed contrary roles in the HBV antibody response (37). In the present study, DQB1^*^05:01 showed a positive response association, and DQB1^*^05:02 showed a negative response association with IJEV, though the association was not significant after FDR correction. Further HLA residue association study indicated that the *DQB1* residue S57, present in DQB1^*^05:02 and DQB1^*^05:04, showed an opposite JEV-NAb response from residue V57, present in DQB1^*^05:01, DQB1^*^06:04, and DQB1^*^06:09. The DQ peptide prediction suggested that the peptide FLVHREWFHDLALPW showed strong binding in both DQA1^*^01:01-DQB1^*^05:01 and DQA1^*^01:01-DQB1^*^05:02, while the peptides HREWFHDLALPWTPP and RNRELLMEFEEAHAT showed strong binding in subjects with DQA1^*^01:01-DQB1^*^05:02, but not in DQA1^*^01:01-DQB1^*^05:01 (Supplementary Table 2). This finding indicates that DQB1^*^05:01 and DQB1^*^05:02 may produce different JEV peptides. Moreover, these data indicate that allele differences may change the binding groove of the antigen-HLA complex, in turn influencing T cell receptors expressed on inactivated JEV-specific CD4^+^ T cells and, finally, playing different roles in the antibody response (27, 37).

In addition to DQB1^*^02:01 and DQB1^*^02:02, other HLA class II genes are reportedly associated with the antibody response to vaccines. For example, Jafarzadeh et al. (27) reported that DRB1^*^01:01, DRB1^*^13:01, DRB1:15:01, and DQB1^*^04:01 were positively associated with HBV antibody response, while DRB1^*^03:01, DRB1^*^07:01, and DQB1^*^02:01 were negatively associated. However, other than DQB1^*^02:01, no HLA alleles have been associated with IJEV in the present study. One of the reasons for different HLA alleles being associated with antibody responses could be a distinct immune response to different vaccines or pathogens. Most previous association studies have been performed with attenuated vaccines (mumps, measles, rubella vaccine, etc.) or virus-like particle-based vaccines (HBV), which could induce both HLA class I- and class II-mediated immune response to generate an immune response. However, in an inactivated vaccine like IJEV, the humoral immune response mediated by HLA class II molecules would be key in generating JEV-NAbs. Thus, the difference in the immune response mechanism between the inactivated vaccine and the attenuated vaccine may be caused by the association with different HLA alleles (18, 38). Another reason could be a population-specific difference in HLA distribution, as with HLA genes and their motifs, even if the populations were administered the same vaccine. For example, in 2015, Jafarzadeh et al. (27) reported that non-responsiveness to HBV is associated with *HLA-A1, -B15*, and *-B40* in Indians, *HLA-A1, -A2*, and *-B8* in Caucasians, *HLA-B54* in Chinese, and *HLA-A10* and *-Cw4* in Turkish people. In addition, DQA1^*^05:01-DQB1^*^02:01 is predominant in Europe, Southwest Asia, and North Africa with frequencies of 19.1, 17.2, and 17.0%, respectively, while its frequency is only 2.7% in North America, and it has not been identified in South America. DQA1^*^02:01-DQB1^*^02:01 is predominant in North Africa, common in European and Southwest Asia, and rare in North Africa and South America (39). To the best of our knowledge, this HLA allele diversity was generated in the long evolutionary interaction between hosts and pathogens, which makes it encode adequate products to generate immune responses against different pathogens. Thus, different HLA alleles were formed as an outcome of specific pathogen infections and are therefore associated with different infectious diseases (40–42). As such, the mechanism of the immune response to different vaccine antigens could vary based on different vaccines.

In addition to the seroconversion rate, GMT is an important factor in evaluating vaccine efficacy. In the present study, we evaluated the relationship between GMTs and HLA alleles. Interestingly, we found that the HLA alleles associated with an antibody response were different from the HLA alleles associated with GMTs. These results indicate that HLA alleles have different roles in the host immune response.

In summary, we investigated the association between HLA class II genes and antibody response after IJEV administration, determining that HLA-DQB1^*^02:01 and HLA-DQB1^*^02:02 were associated with NAb seroconversion. Furthermore, certain *HLA-DRB1* and *-DPB1* alleles were associated with higher GMTs than others. The present study suggests that HLA class II genes may influence the antibody response to IJEV. However, as only 161 individuals were examined in the present study, future studies should comprehensively analyze larger samples.

## Ethics Statement

This study was carried out in accordance with the recommendations of the ethical standards of the Responsible Committee on Human Experimentation of the Ethics Committee of the Guangxi Centre for Disease Control and Prevention, with written informed consent obtained from all subjects in accordance with the Declaration of Helsinki. The protocol was approved by the Inner Mongolia Autonomous Region Center for Disease Control and Prevention.

## Author Contributions

MS and LiS: conceived and designed the experiments. YY, HY, LeS, SL, and CL: performed the experiments. YY, JC, and ZZ: data analysis. MS and LiS: manuscript writing.

### Conflict of Interest Statement

The authors declare that the research was conducted in the absence of any commercial or financial relationships that could be construed as a potential conflict of interest.
