# Supplementary material for: HLA Class II Genes HLA-DRB1, HLA-DPB1, and HLA-DQB1 Are Associated With the Antibody Response to Inactivated Japanese Encephalitis Vaccine
Source: Front Immunol. 2019 Mar 8;10:428. doi: 10.3389/fimmu.2019.00428 (PMC6418001; doi:10.3389/fimmu.2019.00428)
Supplement: Supplementary file 1 [file Table_1.DOCX]

**Supplementary Table 1. HLA Residues Associated with JEV Antibody Seroconversion**

|  |  |  |  |  |  |  |  |
| --- | --- | --- | --- | --- | --- | --- | --- |
| ID | A_pos | B_pos | A_neg | B_neg | P | OR | ACR |
| DPB1_56_A | 120 | 41 | 178 | 34 | 0.027 | 1.78 | DPB1*01:01, DPB1*02:02, DPB1*04:01, DPB1*05:01, DPB1*13:01, DPB1*21:01, DPB1*38:01, DPB1*47:01 |
| DPB1_96_R | 22 | 139 | 49 | 163 | 0.024 | 1.88 | DPB1*02:02, DPB1*17:01 |
| DPB1_170_T | 22 | 139 | 49 | 163 | 0.024 | 1.88 | DPB1*02:02, DPB1*17:01 |
| DPB1_205_V | 23 | 138 | 52 | 160 | 0.019 | 1.93 | DPB1*02:02, DPB1*17:01, DPB1*21:01 |
| DQB1_57_S | 8 | 153 | 24 | 188 | 0.039 | 2.35 | DQB1*05:02, DQB1*05:04 |
| DQB1_57_V | 35 | 126 | 26 | 186 | 0.016 | 0.51 | DQB1*05:01, DQB1*06:04, DQB1*06:09 |
| DQB1_116_V | 4 | 157 | 43 | 169 | 5.93E-08 | 8.98 | DQB1*02:02 |
| DQB1_125_A | 4 | 157 | 43 | 169 | 5.93E-08 | 8.98 | DQB1*02:02 |
| DQB1_135_G | 4 | 157 | 43 | 169 | 5.93E-08 | 8.98 | DQB1*02:02 |
| DQB1_146_P | 4 | 157 | 43 | 169 | 5.93E-08 | 8.98 | DQB1*02:02 |
| DRB1_11_L | 18 | 143 | 7 | 205 | 0.0032 | 0.28 | DRB1*01:01, DRB1*01:02, DRB1*01:04 |
| DRB1_12_K | 143 | 18 | 170 | 42 | 0.032 | 0.52 | DRB1*01:01, DRB1*01:02, DRB1*01:04, DRB1*04:01, DRB1*04:02, DRB1*04:03, DRB1*04:04, DRB1*04:05, DRB1*04:06, DRB1*04:07, DRB1*04:14, DRB1*04:77, DRB1*07:01, DRB1*09:01, DRB1*09:02, DRB1*10:01, DRB1*15:01, DRB1*15:02, DRB1*15:04, DRB1*15:07, DRB1*16:02 |
| DRB1_13_F | 66 | 95 | 58 | 154 | 0.008 | 0.54 | DRB1*01:01, DRB1*01:02, DRB1*01:04, DRB1*09:01, DRB1*09:02, DRB1*10:01 |
| DRB1_26_L | 63 | 98 | 62 | 150 | 0.047 | 0.64 | DRB1*01:01, DRB1*01:02, DRB1*01:04, DRB1*10:01, DRB1*12:01, DRB1*12:02, DRB1*12:21 |
| DRB1_28_D | 121 | 40 | 180 | 32 | 0.024 | 1.85 | DRB1*03:01, DRB1*04:01, DRB1*04:02, DRB1*04:03, DRB1*04:04, DRB1*04:05, DRB1*04:06, DRB1*04:07, DRB1*04:38, DRB1*08:01, DRB1*08:02, DRB1*08:03, DRB1*11:01, DRB1*11:03, DRB1*11:04, DRB1*11:37, DRB1*13:01, DRB1*13:02, DRB1*13:03, DRB1*14:01, DRB1*14:04, DRB1*14:05, DRB1*14:07, DRB1*15:01, DRB1*15:02, DRB1*15:04, DRB1*15:07, DRB1*16:02 |
| DRB1_30_C | 18 | 143 | 7 | 205 | 0.003 | 0.28 | DRB1*01:01, DRB1*01:02, DRB1*01:04 |
| DRB1_30_Y | 125 | 36 | 184 | 28 | 0.026 | 1.88 | DRB1*03:01, DRB1*04:01, DRB1*04:02, DRB1*04:03, DRB1*04:04, DRB1*04:05, DRB1*04:06, DRB1*04:07, DRB1*04:38, DRB1*08:01, DRB1*08:02, DRB1*08:03, DRB1*11:01, DRB1*11:03, DRB1*11:04, DRB1*11:37, DRB1*13:01, DRB1*13:02, DRB1*13:03, DRB1*14:01, DRB1*14:02, DRB1*14:03, DRB1*14:04, DRB1*14:05, DRB1*14:07, DRB1*14:18, DRB1*15:01, DRB1*15:02, DRB1*15:04, DRB1*15:07, DRB1*16:02 |
| DRB1_31_I | 59 | 102 | 52 | 160 | 0.012 | 0.56 | DRB1*01:01, DRB1*01:02, DRB1*01:04, DRB1*09:01, DRB1*09:02 |
| DRB1_32_Y | 152 | 9 | 187 | 25 | 0.046 | 0.46 | DRB1*01:01, DRB1*01:02, DRB1*01:04, DRB1*04:01, DRB1*04:02, DRB1*04:03, DRB1*04:04, DRB1*04:05, DRB1*04:06, DRB1*04:07, DRB1*04:14, DRB1*04:77, DRB1*07:01, DRB1*08:01, DRB1*08:02, DRB1*08:03, DRB1*09:01, DRB1*09:02, DRB1*11:01, DRB1*11:03, DRB1*11:04, DRB1*11:37, DRB1*13:03, DRB1*15:01, DRB1*15:02, DRB1*15:04, DRB1*15:07, DRB1*16:02 |

ID: gene, position and residue OR: the odds ratio calculated with Haldane's correction of Woolf's method ACR: lists the alleles where the residue is present

A_pos: the number of Sero-positive carry the residue at this position; B_pos: the number of Sero-positive do not carry the residue at this position

A_neg: the number of Sero-negative carry the residue at this position; B_neg: the number of Sero-negative do not carry the residue at this position

**Supplementary Table 2. HLA DQ predicted using NetMHCIIpan**

| Allele | Peptide | Seq | Pos | Core | Core_Rel | 1-log50k(aff) | Affinity(nM) | %Rank | Binding Level |
| --- | --- | --- | --- | --- | --- | --- | --- | --- | --- |
| HLA-DQA1*01:01-DQB1*05:01 | FLVHREWFHDLALPW | 211 | 4 | REWFHDLAL | 0.26 | 0.505 | 212.42 | 1 | <=SB |
|  | RNRELLMEFEEAHAT | 234 | 3 | ELLMEFEEA | 0.4 | 0.47 | 308.45 | 2.5 | <=WB |
|  | HREWFHDLALPWTPP | 214 | 3 | WFHDLALPW | 0.335 | 0.461 | 339.46 | 3 | <=WB |
|  | SGLNTEAFYVMTVGS | 194 | 4 | TEAFYVMTV | 0.56 | 0.411 | 588.23 | 6.5 | <=WB |
|  | GASGATWVDLVLEGD | 14 | 3 | GATWVDLVL | 0.445 | 0.397 | 682.77 | 8.5 | <=WB |
| HLA-DQA1*01:01-DQB1*05:02 | FLVHREWFHDLALPW | 211 | 3 | HREWFHDLA | 0.305 | 0.331 | 1386.08 | 0.3 | <=SB |
|  | HREWFHDLALPWTPP | 214 | 1 | REWFHDLAL | 0.27 | 0.28 | 2424.83 | 1.4 | <=SB |
|  | RNRELLMEFEEAHAT | 234 | 3 | ELLMEFEEA | 0.355 | 0.277 | 2498.31 | 1.5 | <=SB |
|  | GLNTEAFYVMTVGSK | 195 | 3 | TEAFYVMTV | 0.53 | 0.218 | 4718.77 | 7 | <=WB |
|  | GSKSFLVHREWFHDL | 207 | 1 | SKSFLVHRE | 0.29 | 0.208 | 5266.97 | 9 | <=WB |
|  | GASGATWVDLVLEGD | 14 | 3 | GATWVDLVL | 0.365 | 0.202 | 5638.57 | 10 | <=WB |
| HLA-DQA1*01:02-DQB1*05:01 | TLDVRMINIEASQLA | 40 | 3 | VRMINIEAS | 0.56 | 0.714 | 22.11 | 0.2 | <=SB |
|  | VRMINIEASQLAEVR | 43 | 3 | INIEASQLA | 0.275 | 0.691 | 28.34 | 0.7 | <=SB |
|  | ATGGVLLFLATNVHA | 486 | 5 | LLFLATNVH | 0.33 | 0.66 | 39.43 | 2.5 | <=WB |
|  | GALLLWMGVNARDRS | 465 | 3 | LLWMGVNAR | 0.37 | 0.66 | 39.62 | 2.5 | <=WB |
|  | RSIALAFLATGGVLL | 478 | 2 | IALAFLATG | 0.37 | 0.65 | 44.24 | 3.5 | <=WB |
|  | SIALAFLATGGVLLF | 479 | 5 | FLATGGVLL | 0.22 | 0.649 | 44.49 | 3.5 | <=WB |
|  | VGRLVTVNPFVATSS | 351 | 3 | LVTVNPFVA | 0.445 | 0.648 | 45.03 | 3.5 | <=WB |
|  | LATGGVLLFLATNVH | 485 | 5 | VLLFLATNV | 0.395 | 0.624 | 58.14 | 6.5 | <=WB |
|  | GLMGALLLWMGVNAR | 462 | 5 | LLLWMGVNA | 0.365 | 0.622 | 59.66 | 6.5 | <=WB |
|  | KIPIVSVASLNDMTP | 336 | 3 | IVSVASLND | 0.475 | 0.607 | 70.19 | 9 | <=WB |
|  | GLHQALAGAIVVEYS | 261 | 3 | QALAGAIVV | 0.23 | 0.605 | 71.95 | 9.5 | <=WB |
|  | LHQALAGAIVVEYSS | 262 | 4 | LAGAIVVEY | 0.32 | 0.603 | 73.11 | 9.5 | <=WB |
| HLA-DQA1*01:02-DQB1*05:02 | RNRELLMEFEEAHAT | 234 | 3 | ELLMEFEEA | 0.415 | 0.473 | 300.23 | 1 | <=SB |
|  | FLVHREWFHDLALPW | 211 | 4 | REWFHDLAL | 0.25 | 0.466 | 324.12 | 1.2 | <=SB |
|  | KSFLVHREWFHDLAL | 209 | 5 | HREWFHDLA | 0.285 | 0.404 | 633.38 | 5 | <=WB |
|  | ASGATWVDLVLEGDS | 15 | 2 | GATWVDLVL | 0.315 | 0.386 | 769.1 | 7 | <=WB |
|  | SGLNTEAFYVMTVGS | 194 | 4 | TEAFYVMTV | 0.535 | 0.382 | 805.27 | 7.5 | <=WB |
|  | ALLLWMGVNARDRSI | 466 | 3 | LWMGVNARD | 0.325 | 0.376 | 859.36 | 8.5 | <=WB |
|  | REWFHDLALPWTPPS | 215 | 2 | WFHDLALPW | 0.355 | 0.375 | 867.7 | 8.5 | <=WB |
|  | TLDVRMINIEASQLA | 40 | 3 | VRMINIEAS | 0.45 | 0.374 | 873.37 | 8.5 | <=WB |
|  | GALLLWMGVNARDRS | 465 | 3 | LLWMGVNAR | 0.33 | 0.373 | 881 | 9 | <=WB |
| HLA-DQA1*05:01-DQB1*02:01 | GLHQALAGAIVVEYS | 261 | 5 | LAGAIVVEY | 0.485 | 0.513 | 193.67 | 1.2 | <=SB |
|  | VHREWFHDLALPWTP | 213 | 4 | WFHDLALPW | 0.53 | 0.497 | 231.96 | 1.7 | <=SB |
|  | RNRELLMEFEEAHAT | 234 | 3 | ELLMEFEEA | 0.425 | 0.489 | 251.5 | 2.5 | <=WB |
|  | GASGATWVDLVLEGD | 14 | 4 | ATWVDLVLE | 0.35 | 0.472 | 303.74 | 3.5 | <=WB |
|  | DVRMINIEASQLAEV | 42 | 3 | MINIEASQL | 0.27 | 0.456 | 361.7 | 4.5 | <=WB |
|  | VRMINIEASQLAEVR | 43 | 5 | IEASQLAEV | 0.255 | 0.441 | 423.29 | 6 | <=WB |
|  | ATWVDLVLEGDSCLT | 18 | 2 | WVDLVLEGD | 0.33 | 0.437 | 441.9 | 6 | <=WB |
|  | SSANSKVLVEMEPPF | 364 | 3 | NSKVLVEME | 0.405 | 0.424 | 508.23 | 8 | <=WB |
|  | GAQRLAALGDTAWDF | 413 | 4 | LAALGDTAW | 0.275 | 0.42 | 534.2 | 8.5 | <=WB |
|  | ANSKVLVEMEPPFGD | 366 | 4 | VLVEMEPPF | 0.28 | 0.409 | 597.72 | 10 | <=WB |
|  | PTLDVRMINIEASQL | 39 | 4 | VRMINIEAS | 0.385 | 0.407 | 613.86 | 10 | <=WB |
| HLA-DQA1*05:01-DQB1*02:02 | GLHQALAGAIVVEYS | 261 | 5 | LAGAIVVEY | 0.485 | 0.513 | 193.67 | 1.2 | <=SB |
|  | VHREWFHDLALPWTP | 213 | 4 | WFHDLALPW | 0.53 | 0.497 | 231.96 | 1.7 | <=SB |
|  | RNRELLMEFEEAHAT | 234 | 3 | ELLMEFEEA | 0.425 | 0.489 | 251.5 | 2.5 | <=WB |
|  | GASGATWVDLVLEGD | 14 | 4 | ATWVDLVLE | 0.35 | 0.472 | 303.74 | 3.5 | <=WB |
|  | DVRMINIEASQLAEV | 42 | 3 | MINIEASQL | 0.27 | 0.456 | 361.7 | 4.5 | <=WB |
|  | VRMINIEASQLAEVR | 43 | 5 | IEASQLAEV | 0.255 | 0.441 | 423.29 | 6 | <=WB |
|  | ATWVDLVLEGDSCLT | 18 | 2 | WVDLVLEGD | 0.33 | 0.437 | 441.9 | 6 | <=WB |
|  | SSANSKVLVEMEPPF | 364 | 3 | NSKVLVEME | 0.405 | 0.424 | 508.23 | 8 | <=WB |
|  | GAQRLAALGDTAWDF | 413 | 4 | LAALGDTAW | 0.275 | 0.42 | 534.2 | 8.5 | <=WB |
|  | ANSKVLVEMEPPFGD | 366 | 4 | VLVEMEPPF | 0.28 | 0.409 | 597.72 | 10 | <=WB |
|  | PTLDVRMINIEASQL | 39 | 4 | VRMINIEAS | 0.385 | 0.407 | 613.86 | 10 | <=WB |
| HLA-DQA1*02:01-DQB1*02:01 | WRNRELLMEFEEAHA | 233 | 4 | ELLMEFEEA | 0.44 | 0.392 | 720.11 | 1.8 | <=SB |
|  | GLHQALAGAIVVEYS | 261 | 5 | LAGAIVVEY | 0.46 | 0.381 | 813.31 | 2.5 | <=WB |
|  | GASGATWVDLVLEGD | 14 | 4 | ATWVDLVLE | 0.385 | 0.375 | 863.91 | 3 | <=WB |
|  | LVHREWFHDLALPWT | 212 | 5 | WFHDLALPW | 0.45 | 0.361 | 1007.72 | 4 | <=WB |
|  | DVRMINIEASQLAEV | 42 | 3 | MINIEASQL | 0.23 | 0.354 | 1085.19 | 4.5 | <=WB |
|  | VRMINIEASQLAEVR | 43 | 5 | IEASQLAEV | 0.25 | 0.345 | 1201.21 | 5 | <=WB |
|  | ATWVDLVLEGDSCLT | 18 | 2 | WVDLVLEGD | 0.31 | 0.337 | 1306.64 | 6 | <=WB |
|  | SSANSKVLVEMEPPF | 364 | 3 | NSKVLVEME | 0.415 | 0.327 | 1454.65 | 7 | <=WB |
|  | PTLDVRMINIEASQL | 39 | 4 | VRMINIEAS | 0.405 | 0.312 | 1706.39 | 9.5 | <=WB |
|  | IPIVSVASLNDMTPV | 337 | 2 | IVSVASLND | 0.365 | 0.31 | 1754.2 | 9.5 | <=WB |
| HLA-DQA1*02:01-DQB1*02:02 | WRNRELLMEFEEAHA | 233 | 4 | ELLMEFEEA | 0.44 | 0.392 | 720.11 | 1.8 | <=SB |
|  | GLHQALAGAIVVEYS | 261 | 5 | LAGAIVVEY | 0.46 | 0.381 | 813.31 | 2.5 | <=WB |
|  | GASGATWVDLVLEGD | 14 | 4 | ATWVDLVLE | 0.385 | 0.375 | 863.91 | 3 | <=WB |
|  | LVHREWFHDLALPWT | 212 | 5 | WFHDLALPW | 0.45 | 0.361 | 1007.72 | 4 | <=WB |
|  | DVRMINIEASQLAEV | 42 | 3 | MINIEASQL | 0.23 | 0.354 | 1085.19 | 4.5 | <=WB |
|  | VRMINIEASQLAEVR | 43 | 5 | IEASQLAEV | 0.25 | 0.345 | 1201.21 | 5 | <=WB |
|  | ATWVDLVLEGDSCLT | 18 | 2 | WVDLVLEGD | 0.31 | 0.337 | 1306.64 | 6 | <=WB |
|  | SSANSKVLVEMEPPF | 364 | 3 | NSKVLVEME | 0.415 | 0.327 | 1454.65 | 7 | <=WB |
|  | PTLDVRMINIEASQL | 39 | 4 | VRMINIEAS | 0.405 | 0.312 | 1706.39 | 9.5 | <=WB |
|  | IPIVSVASLNDMTPV | 337 | 2 | IVSVASLND | 0.365 | 0.31 | 1754.2 | 9.5 | <=WB |

Allele: MHC molecule name; Peptide: Amino acid sequence; Seq: Residue number; Pos: Starting position of the optimal binding core

Core: Binding core register; Core_Rel: Reliability of the binding core, expressed as the fraction of networks in the ensemble selecting the optimal core

 1-log50K(aff): Predicted binding affinity in log-scale; Affinity(nM): Predicted binding affinity in nanomolar IC50

SB: Strong binding; WB: Weak binding
